# Fractionation of Waste MDF by Steam Refining

**DOI:** 10.3390/molecules25092165

**Published:** 2020-05-05

**Authors:** Sebastian Hagel, Bodo Saake

**Affiliations:** Institute of Wood Science, Chemical Wood Technology, Universität Hamburg, Haidkrugsweg 1, 22885 Barsbüttel, Germany; Sebastian.Hagel@uni-hamburg.de

**Keywords:** biorefinery, MDF, recycling, waste valorization, steam treatment, hemicelluloses, fibers

## Abstract

In view of the expected increase in available waste medium-density fiberboard (MDF) and the current insufficient and unsatisfactory disposal capacities, efficient ways of recycling the waste material need to be developed. In this study, the potential of steam refining as a method to hydrolyze the resins, isolate fibers, and obtain a hemicellulose-rich extract available for further utilization in the context of a biorefinery was assessed. Two different MDF waste samples, as well as poplar (*Populus spp.*) and spruce (*Picea spp.*) wood chips for benchmarking, were treated over a severity range from 2.47 to 3.95. The separated fiber and extract fractions were analyzed with regard to yield, content of carbohydrates, acids, degradation products, and nitrogen. A fiber fraction of more than 70% yield and an extract containing up to 30% of carbohydrates for further processing can be gained by steam-refining waste MDF. At low severities, most of the nitrogen-based compounds are solubilized. Increasing the severity leads to a decrease in nitrogen in the extract as the nitrogen compounds are converted into volatiles. A non-hydrolysable resin residue remains on the fibers, independent of the treatment severity. In comparison to the benchmark samples, the extract fraction of waste MDF shows a high pH of 8 and high amounts of acetic and formic acid. The generation of furfural and 5-hydroxymethylfurfural (5-HMF) on the other hand is suppressed. Distinct differences in carbohydrate hydrolysis behavior between waste MDF and conventional wood can be observed. Especially, the mannose-containing constituents seem to be resistant to hydrolysis reactions in the milieu created in MDF fractionation.

## 1. Introduction

The development of engineered wood was a significant achievement for the wood-working industry, allowing previously underused low-grade wood sources to be valorized and enabling the manufacturing of wood-based materials with more uniform properties [1]. Medium-density fiberboard (MDF), a panel product made by hot-pressing refined wood fibers blended with resins, has been a tremendous success worldwide [2]. Softwood, hardwood, recycled wood, and even annual plants can be processed as raw material for MDF production [1,2,3,4]. Currently, roughly 80% of the annual production is processed for furniture or flooring applications [5]. The first commercial plant began production of MDF in 1965 in Deposit, NY, USA, under the trade name ‘Baraboard’ [4]. Since then, the amount of MDF manufactured globally has risen steadily, with roughly 100 million m^3^ produced in 2018 [6]. However, while, from a commercial point of view, the history of MDF can be considered a great success, there is still a big void in viable and commercially successful recycling methods, and a major part of the waste MDF is either burned for energy use or, if insufficient capacity is available, put into landfills [7,8]. Following the principles of the European Waste Hierarchy Directive [9], reuse and recycling methods should be given priority over energy recovery and disposal methods, as previously mentioned strategies put a huge strain on the environment through pollution of air, water, and soil [10]. It is estimated that 25% of the produced MDF is converted to waste in the form of off-cuts, machining errors, or transport and storage losses within one year, and 99% of the currently produced MDF can be considered waste within 45 years. Using an average life span of 14 years, close to 50 million m^3^ of waste MDF was accrued worldwide in 2016 alone, with this tendency rising [8].

Taking this huge amount of lignocellulosic waste and the global endeavor to conserve natural resources into account, the urgency for viable recycling methods of this so-far untapped and ubiquitous material source becomes apparent. Biorefineries, in which all bio-based material components are valorized, are considered to play a key role in the transformation of the current fossil-based economy to a sustainable bioeconomy [11]. Waste MDF might be an economically attractive source of lignocellulosic material for such a biorefinery. In biorefineries, as a first step, the raw material is typically fractionated to render the individual components available for further processing [12,13]. Thus, a thorough understanding of the fractionation behavior of all components, including the adhesive systems, is crucial in valorization of waste MDF.

The most used adhesive system in the production of MDF is urea-formaldehyde (UF), either exclusively or fortified with a small amount of melamine. UF-resin systems with a higher amount of melamine of 12% or more are less common. Phenol formaldehyde resin (PF) and polymeric methylene diphenyl diisocyanate (PMDI) adhesive systems are used only in miniscule amounts [14]. Depending on the desired application, additives can be utilized, such as fungicides and water-repellant or fire protection agents [2,14]. The main advantages of UF-based adhesives are their low cost, high reactivity, and translucent color. At the same time, due to their susceptibility to hydrolysis and thus weak moisture resistance, they are considered unsuitable for outdoor use [15]. However, this susceptibility to hydrolysis can be used in the fractionation of MDF for recycling purposes. While the highest removal rate of cured resins can be achieved using acidic solutions [16], it is still possible to remove up to two-thirds of cured resin using only water at temperatures below 100 °C [17]. More intense reaction conditions, as they are found in steam treatments, can further improve the resin hydrolysis [17,18,19,20].

Pure steam treatments can be considered auto-hydrolytic processes, as water is the only reagent added [21,22]. In comparison to treatments with acid, no recovery steps are needed [23] and less corrosion-resistant material is required [24,25], leading to a comparatively low operating cost and environmental impact [26]. Steam refining, a process similar to steam explosion [27], utilizes high-pressured steam at elevated temperatures to induce auto-hydrolysis in the wood with a subsequent refining step at the end of the steam treatment to open the structure mechanically. The severity factor, introduced by Overend et al. [28], combines the reaction duration and temperature into a single parameter and can be used to describe and compare changes in the main polymers of lignocellulosic material in steam treatments.

Steam treatments of lignocellulosic material have been utilized successfully in industry [29] as a pretreatment method for enzymatic hydrolysis with subsequent microbial fermentation to gain ethanol [27,30,31] or platform chemicals like succinic acid or lactic acid [32,33]. In such pretreatments for enzymatic hydrolysis, the goal is to open up the dense structure of the lignocellulosic material and increase the enzyme digestibility of the cellulose [34]. However, steam treatments can also be used to extract oligosaccharides [21,35,36,37]. Lately, there has been a growing interest in utilizing oligosaccharides in different applications such as biodegradable oxygen barrier films [38,39,40], emulsifiers [41,42,43,44,45,46,47], thermoplastics [48], hydrogels [49], coatings, or adhesives [50]. The remaining fibrous fraction, on the other hand, might be suitable for production of new fiberboards [51,52,53,54,55,56,57], cellulose nanocrystals [58,59], bioethanol [60,61], biogas, and bio-oil using pyrolysis [62,63]; however, utilization as reinforcement and filler material in wood–plastic compounds (WPCs) [64,65,66] and particleboards [67] has also been proposed.

Depending on the wood species and treatment conditions used, extracts of hydrothermally treated wood usually display a pH in the range from 3 to 5 [68,69,70]. Due to hydrolyzed resin compounds, the extracts of waste MDF can display a pH value of up to 9 [20,71]. The changed milieu is likely to influence the hydrolysis reactions of the lignocellulosic material and thus influence the properties of the waste MDF fractions. As these properties play a key role in the further processing and final product properties, the aim of the present study was: (i) To characterize and compare the resulting fractions of steam-refined post-consumer MDF and conventionally utilized wood species over a wide range of treatment severities by determining the content of carbohydrates, lignin, acids, and degradation products in addition to yield rate; (ii) to verify the applicability of the severity factor concept for waste MDF; (iii) to evaluate the viability of low-severity steam refining as a process to hydrolyze cured resins of post-consumer MDF by determining the nitrogen content of the fiber and extract fractions; (iv) to crosslink the abovementioned information to broaden the knowledge on the influence of the resins on the auto-hydrolytic reactions of the wood components and vice versa.

Because of their widespread availability and utilization in industry, poplar and spruce wood chips (as representatives for hard- and softwood, respectively) were evaluated in addition to two different MDF samples and compared to each other. For every material, steaming experiments with 10 differing temperature and time combinations were performed. Evaluating temperatures between 150 and 190 °C in increments of 10 °C and durations of 10 and 20 min, a severity range from 2.47 to 3.95 was covered, and, by using different time–temperature combinations, resulting in the same severity grade, the assumed interchangeability was verified for MDF.

## 2. Materials and Methods

### 2.1. Raw Materials and Their Characterization

Both MDF charges were supplied by École supérieure du bois (Nantes, France). The first MDF charge (MDF A) consisted of off-cuts of standard panels from the in-house workshop, while the second charge (MDF B) was collected directly from an MDF producer. The MDF samples of 10 mm thickness were sawn into strips of 15 mm width and, afterward, passed through a chipper, creating chips with a length of 10 to 50 mm. Wood chips of poplar (*Populus spp.*) and spruce (*Picea spp.*) were supplied by Lanaken Mill (Sappi, Belgium). To achieve a more homogeneous fraction for experimentation, the wood chips were screened, and fine, oversized chips and bark residuals were removed by hand. The accepted fraction of wood chips featured a thickness of no less than 8 mm and a length of about 20 to 30 mm. The moisture content of the wood chips was 45–55%. To prevent biological degradation, the chips were stored at −20 °C until the day before the experiments were carried out.

The ash content was determined according to TAPPI T 211 om-16 and the elemental composition was determined using the Dumas-method with a vario EL cube (Elementar, Langenselbold, Germany). Extractive content was determined with an ASE 350 (Dionex, Sunnyvale, CA, USA) on milled (SM 2000, Retsch, Haan, Germany) material with a size of ≤1 mm diameter. The extraction was performed in three succeeding steps (petroleum ether (70 °C); acetone/water 9:1 (70 °C); water (90 °C)) with a pressure of 10 MPa. The carbohydrate and lignin content determination were carried out on extracted material after acid hydrolysis and is described in detail in Section 2.4.

### 2.2. Steam-Refining Treatment and Experimental Plan

The steam refining was carried out on 300 g of dry material in a cylindrical 10 L reactor by Martin Busch & Sohn GmbH (Schermbeck, Germany) with a diameter of 22 cm and a length of 25 cm for every experimental run. The severity factor ($R_{0}$), combining the reaction duration (*t* in minutes) and temperature (*T* in °C), was calculated according to Equation (1).

(1) $$logR_{0}=t\times e^{\frac{\left(T-100\right)}{14.75}}$$

The experiments were carried out using temperatures varying between 150 °C and 190 °C and durations of 10 and 20 min, as summarized in Table 1. The reactor was equipped with a four-bladed system, which was rotated at approximately 1455 rpm by an electric motor with a power of 6.8 kW for 30 s at the end of each steaming treatment. Afterward, the steam pressure was released through a valve in about 90 s. The content of the reactor was transferred into a tank by flushing with roughly 10 L of water. The fiber suspension was filtered through a sieve bag and the extract was separated by centrifugation in a spin-dryer (Thomas Centri 776 SEK, Thomas, Neunkirchen, Germany) for 10 min at 2800 rpm. The newly formed fiber fraction was homogenized for 10 min in a rotary stirrer with a 20 L volume (Hobart A20, Hobart, Offenburg, Germany) and the total weight was recorded. Subsequently, the fibrous material was filled into a PE-bag and stored until further evaluation at −20 °C. The extract fraction was weighted, and the pH was determined with a pH-Meter ph 330i (WTW, Weilheim, Germany). Dry matter contents were determined by freeze-drying the extracts with an Alpha 2-4 LSC (Martin Christ Gefriertrocknungsanlagen GmbH, Osterode am Harz, Germany) and drying part of the fibers at 105 °C to calculate the yields based on raw material input.

### 2.3. Acid Hydrolysis of Steam-Refined Fibers and Extract Fractions

To determine the monomeric carbohydrate content of the fibers and the raw substrate, the material was air-dried and milled to fine powder with a T-1000 disc mill (Siebtechnik GmbH, Mülheim an der Ruhr, Germany). Subsequently, 2-stage acid hydrolysis was carried out by mixing 200 mg of dry material with 2 mL of sulfuric acid (72%, Fisher Scientific, Hampton, NH, USA) and pre-hydrolyzing at 30 °C. After 60 min, the reaction was stopped by adding 6 mL deionized water, and the sample was transferred with 50 mL deionized water into a 100 mL flask [72]. For the extracts, 100 mg of lyophilized sample was dissolved in 10 mL water and hydrolyzed in 1.8 mL 2N H_2_SO_4_. Subsequently, post-hydrolysis was carried out for 40 min (30 min for hardwood samples) in an autoclave at 120 °C and 0.12 MPa on all samples to obtain monomeric sugars. Afterward, the hydrolyzed samples of the fibers and extracts were cooled and filtered on a G4 sintered glass crucible with distilled water. The acid-insoluble residue (analogous to Klason–Lignin) was dried at 105 °C and determined gravimetrically. All samples were hydrolyzed in triplicate.

### 2.4. Analytical Work

From the hydrolyzed filtrates, the carbohydrate content was determined with a Dionex Ultimate 3000 (Dionex, Sunnyvale, CA, USA) using Borate-HPAEC as described by Lorenz et al. [72]. The acid-soluble lignin content was determined with a UV-Spectrophotometer LAMBDA 650 (PerkinElmer, Waltham, MA, USA) at a wavelength of 205 nm as described by Maekawa et al. [73]. Acetic and formic acids were determined from the extracts by ion chromatography, using an Ionpac AS11-HC 2 · 250 mm anion exchange column (Dionex, Sunnyvale, CA, USA) conditioned at 30 °C with 0.38 mL min^−1^ of 1 mM to 70 mM KOH (Dionex, Sunnyvale, CA, USA) and detected by suppressed conductivity. Furfural and 5-hydroxymethylfurfural (5-HMF) were determined directly from the extracts after steam treatment by reverse-phase high-performance liquid chromatography (Jasco, Tokyo, Japan). An Aquasil C18 column (Thermo Scientific, Waltham, MA, USA) was used at 30 °C. A linear gradient was used, starting with 1 mL min^−1^ acetonitrile (Mallinckrodt Baker Bv, Deventer, Netherlands), ending after 80 min with 100% 1 mM phosphoric acid (Riedel-de Haen, Seelze, Germany). Furfural (Merck, Darmstadt, Germany) and 5-HMF (Sigma Aldrich, Steinheim, Germany) were used as substance standards. A wavelength of 280 nm was used for UV-detection. For the measurement of the fiber length (length-weighted) and width of the fibers, a Kajaani FiberLab from Metso (Helsinki, Finland) was used on part of the fibrous material, after three passes through a laboratory refiner, using consecutively smaller gap distances of 0.05 to 0.02 mm and consistencies of 4% to 2%.

## 3. Results and Discussion

### 3.1. Raw Material Characterization

In order to characterize the raw material extractives, the carbohydrate, lignin, and nitrogen contents were determined. In comparison to untreated wood chips of poplar and spruce, high amounts of extracts were found in MDF sample A and B, with 12.5% and 15.8%, respectively (see Table 2). In both samples, the highest amount of material could be found in the water extract, followed by the acetone/water extract. These findings can most likely be attributed to the presence of UF-resins, which are readily hydrolysable in water at temperatures below 100 °C [16,17,74,75]. This assumption is further strengthened by the elemental composition of the samples before and after extraction. A nitrogen content of 4.2% and 4.4% before extraction was measured in MDF A and B, respectively. Roughly two thirds of the total nitrogen of the MDF samples was removable by the accelerated solvent extraction (ASE). The high amount of nitrogen found in the MDF samples before extraction likely originates from UF-resins, as urea is composed of roughly 46.7% nitrogen. From the measured nitrogen content, assuming a molar ratio of urea to formaldehyde of 1:1, a hypothetical resin content of 13.5% for MDF A and 14.1% for MDF B can be calculated.

The differences in extract content determined between sample A and B can be explained with variations in the amount of resin, the type of resin [14], and the curing degree [74]. A similar amount of nitrogen was found in standard MDF (Pfleiderer, Baruth/Mark, Germany) by Kraft [17].

Both MDF samples show a lower total carbohydrate content than the spruce and poplar sample, and comparing the two MDF samples to each other, significant differences in xylose, mannose, and galactose content can be observed. Sample A shows a higher xylose content with 12.4% and lower mannose and galactose content with 4.0% and 0.7% in comparison to sample B with 5.7%, 7.2%, and 1.3%, respectively. Possible reasons for these differences in carbohydrate compositions are manifold, from alterations in the raw material before MDF production through extended periods of storage in moist conditions via degradation processes induced by microbial activity or heat effects [76,77] to partial degradation of hemicelluloses in the MDF production process itself [78,79], but also, the amount of bark and compression- and tension-wood [80] can influence the carbohydrate composition. However, as all kinds of woody raw material can be used for MDF production, the differences in carbohydrate composition between MDF sample A and B most likely originate from differences in wood species used in production. In general, the hemicelluloses of hardwoods mainly consist of glucuronoxylan in combination with small amounts of glucomannan, while softwood primarily consists of galactoglucomannans and arabino-glucuronoxylans [80,81]. This is in accordance with the carbohydrate composition determined for the poplar and spruce wood chips evaluated in this study. The relative high amount of xylose in sample A suggests a high amount of hardwood included in the sample, while the high amount of mannose in Sample B suggests a larger softwood fraction in comparison to sample A. As the amount of mannose determined in sample B is still significantly less than is usually found in softwood [81], a mixture of soft- and hardwood might have been used in the production of MDF sample B. However, for all the above-mentioned reasons, it is impossible to calculate the exact hardwood/softwood ratios for the samples.

### 3.2. Effect of Severity on the Yield of Waste MDF in Comparison to Poplar and Spruce Wood

**Figure 1:**
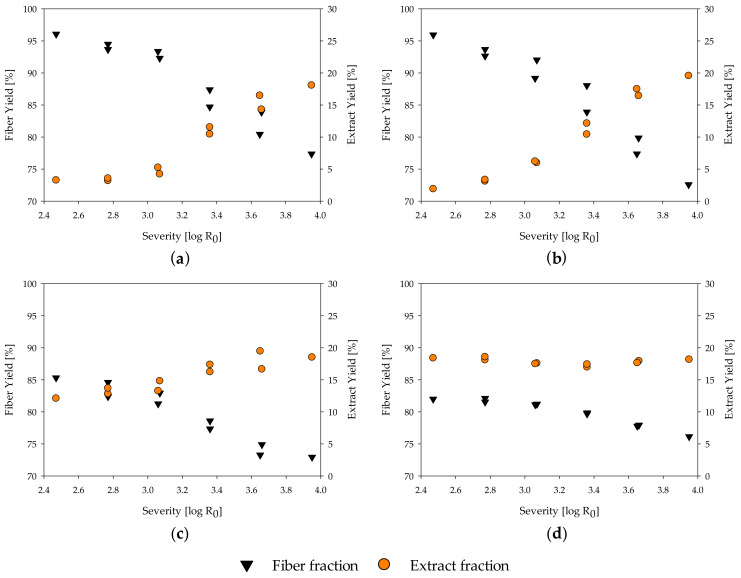
Influence of severity on yield of extract and fiber fraction of (**a**) poplar; (**b**) spruce; (**c**) MDF Sample A; (**d**) MDF Sample B.

The yield after fractionation is an important parameter for evaluating the process reactions and the economic implication. In the evaluated severity range, the fiber yield of all materials decreases with increasing severity (Figure 1a–d, complete data in Table A1). For poplar and spruce wood, a fiber yield of roughly 96% is calculated at a severity of 2.47, and 77.4% and 72.6%, respectively, at a severity of 3.95. At the same time, the extract yield increases from 3.3% for poplar and 1.9% for spruce at a severity of 2.47 to 18.1% and 19.6% at a severity of 3.95 (Figure 1a,b), as hemicelluloses progressively solubilize [21,82]. For poplar wood, a maximum extract yield of 21.4% at a severity of 4.53 was determined by Schütt et al. [83]. At even higher severities, the amount of extract decreases again, as degradation reactions of the mono- and polymers into volatile compounds increase. Thus, the maximum extract yield can be gained at a severity in which the amount of material solubilized is in equilibrium with the amount of mono- and polymers transformed into volatiles by degradation reactions.

In contrast to the benchmarking samples, MDF sample A and B show a much lower fiber yield with 85.3% and 82.0% and a higher extract yield of 12.1% and 18.4%, respectively, at a severity of 2.47 (Figure 1c,d). This matches with the finding of the accelerated solvent extraction performed for raw material characterization, in which a high amount of material was found to be extractable at temperatures below 100 °C. Adding the calculated amount of resins to the fiber yield leads to an adjusted fiber yield of 98.8% and 96.1% for MDF A and B, respectively, which is in line with the fiber yield of native wood. Thus, the high amount of extract and low amount of fiber yield at low severities, in comparison to the native spruce and poplar wood, mainly results from the resins in the MDF samples. 

At a severity of 3.95, all samples reach a similar value of 18% to 20% extract yield and 72% to 78% of fiber yield. The decrease in fiber yield from a severity of 2.47 to 3.95 is lower in the MDF samples than in the native wood samples. As the amount of resin found on the fibers seems to be independent of the treatment severity, this is unlikely to be explainable by changes in the resin residue, but might be due to a deceleration of hydrolysis reactions of the main polymers. This aspect is discussed in detail in Section 3.4. The extract yield of MDF sample B (Figure 1d) seems to be independent of the severity grade. One possible explanation is that the resin degradation in the extract is in equilibrium with the carbohydrate solubilization from the wood material, leading to changes in the composition of the extract but keeping the quantity constant.

### 3.3. Influence of the Treatment Severity on the Presence of Nitrogen Compounds in the Fractions of Waste MDF

The behavior of the resins in steaming treatments is of special interest, as two concurrent hydrolysis reactions are happening in the steam treatment of MDF: The hydrolysis of the resins and the hydrolysis of the wood polymers. It can be expected that the resins and their hydrolysis products influence the hydrolysis reactions of the lignocellulosic material and vice versa. 

**Figure 2:**
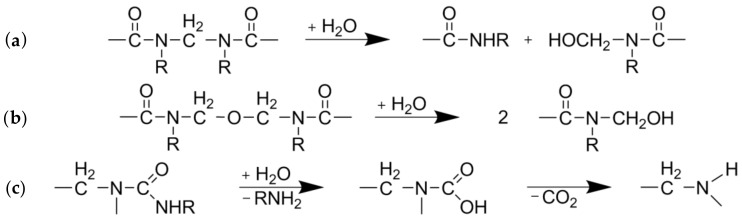
Degradation pathways of urea-formaldehyde (UF)-resin polymers: Cleavage of (**a**) methylene or (**b**) methylene-ether bridges and (**c**) separation of an amine [19].

**Figure 3:**
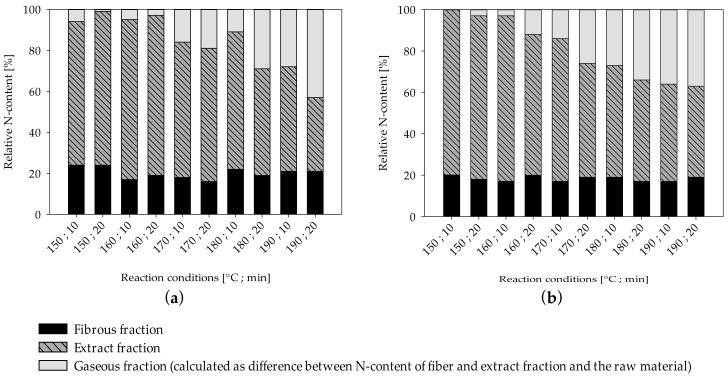
Distribution of total nitrogen content in the fiber, extract, and gaseous fraction (calculated as difference between nitrogen content of fiber and extraction fraction and raw material) of MDF sample A (**a**) and MDF sample B (**b**).

UF-resin polymers are hydrolytically degradable into smaller fragments by cleavage of methylene (Figure 2a) and methylene-ether (Figure 2b) bridges. The resulting terminal hydroxymethyl groups (Figure 2a,b) can undergo dissociation into formaldehyde and urea derivates. Another possible degradation mechanism is by separation of an amine and formation of an intermediate carbamic acid (Figure 2c) [19]. As nitrogen is the main component of UF-resins, it can be measured for quantitative analysis of resin hydrolysis. The nitrogen content of the separate fiber and extract fractions was measured by elemental analyses and put into relation with the corresponding yields and the total nitrogen content found in the raw material to create mass balances (Figure 3). The difference between the total nitrogen content of the raw material and nitrogen found in the extract and fibers combined is assumed to be volatile and transformed into the gaseous phase. From 70% to 80% of the total resin is solubilized at the lowest severity assessed in this study, and roughly 20% of the nitrogen remains on the fibers, independent of the treatment severity.

For both MDF samples, the amount of nitrogen in the extract decreases as the severity rises, while the amount of nitrogen assumed to be in the gaseous phase rises. Using less intense reaction conditions than examined in this study leads to an increasing rate of solubilization of nitrogen compounds, and an ensuing decrease in nitrogen found on fibers can be observed [75]. However, as no change in nitrogen content of the fiber fraction can be observed in this study, the remaining 20% of nitrogen compounds found on the fibers in this study seem to be comparatively resistant to hydrolysis. To evaluate if the nitrogen compounds on the fibers can be easily removed, the nitrogen content of the sample treated at a severity of 3.95 was measured again after the additional refining step, which includes a very effective washing protocol. However, no substantial difference in nitrogen content could be determined. Thus, besides being highly resistant to hydrolysis, the remaining nitrogen compounds in the fiber fraction cannot be removed mechanically and are likely chemically linked to the fibers. By increasing the severity of the treatment, the nitrogen compounds found in the extract, on the other hand, seem to increasingly degrade to volatile products, which pass over from the extract into the gas phase.

Additionally, the resin hydrolysis behavior reported in this study shows distinct differences to the hydrolysis behavior of pure, cured UF resins. In experiments done by Grigsby et al. [74], almost no change in nitrogen content could be observed in pure acid-cured UF resins extracted with water at 60 °C over 24 h. In the MDF samples used in this study, on the other hand, a high amount of material, of which nitrogen is a big part, could be extracted using water at 90 °C (Table 2). In experiments conducted by Fleischer and Marutzky [19], pure UF resin steamed for 50 min at 130 °C released only 14% of the material’s total formaldehyde, while UF-resin-bonded particleboards steamed under the same conditions released up to 70% of the total formaldehyde. A comparable amount of formaldehyde release was determined by Kraft and Roffael [75] using similar treatment conditions on MDF samples. As the formaldehyde release can be used as an indicator for the resin hydrolysis, the much lower formaldehyde release of the pure UF resins indicates a high hydrolysis resistance in comparison to UF resins blended with fibers. One possible reason for this might be the fine distribution of the resin among the fibers in production and its impact on the curing degree of the resin [74]. Interactions between acids released from the fibers, resin, and hardeners might also influence the curing and degradation of the resins. 

### 3.4. Influence of the Severity on the Hydrolysis Reactions of the Wood Polymers

**Figure 4:**
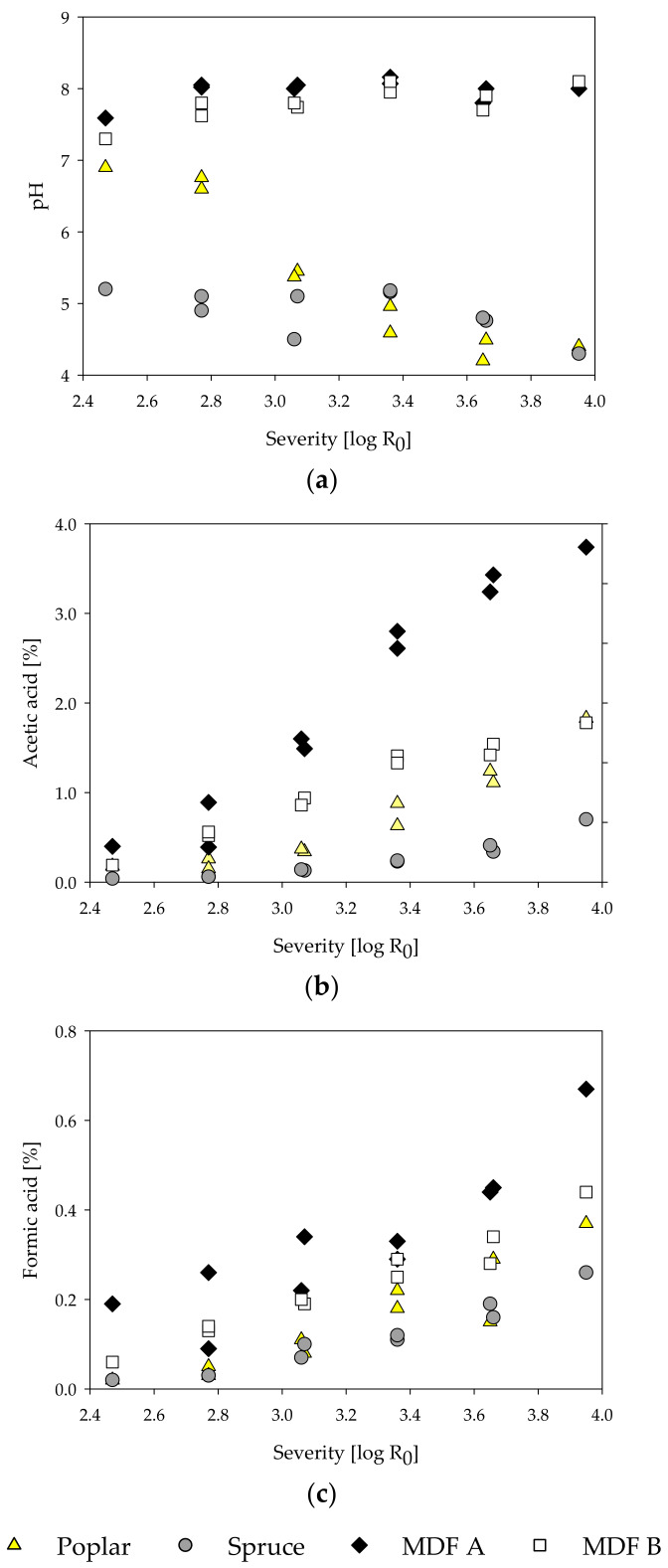
Influence of treatment severity on pH (**a**), and acetic acid (**b**) and formic acid (**c**) contents of steam-refining extracts of poplar, spruce, and the MDF samples.

**Figure 5:**
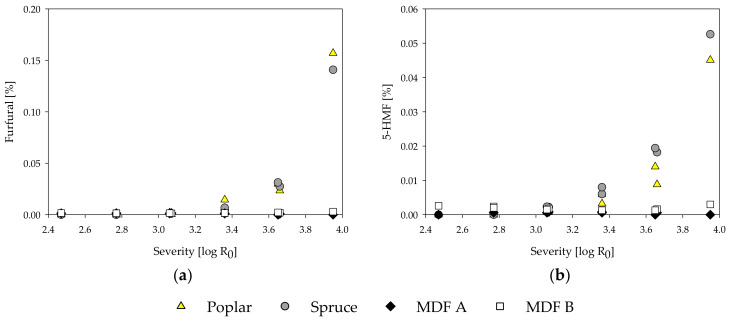
Influence of treatment severity on furfural (**a**) and 5-hydroxymethylfurfural (5-HMF) (**b**) content of steam-refining extracts of poplar, spruce, and the MDF samples.

During the treatment, steam penetrates and condenses inside the microporous structures of the lignocellulosic material. In general, at high temperatures, the condensed water dissociates and creates an acidic milieu [84]. The hydronium ion concentration starts to rise as acetyl groups are cleaved from hemicelluloses by acid-catalyzed hydrolysis, further increasing the reaction rate [85]. As a function of temperature and pH, ester and ether bonds are cleaved and, consequently, carbohydrates are depolymerized and start solubilizing [82]. Concurrently, volatile degradation products like furfural from pentoses and 5-HMF from hexoses are formed [81]. Thus, to characterize the steam-refined fractions in detail and assess the hydrolysis reactions, the content of carbohydrates and acid-insoluble residue (AIR) was determined for the fiber and extract fractions for all samples (Table 3). Additionally, the extracts were examined in regard to pH, acetic and formic acid (Figure 4), and degradation products such as furfural and 5-HMF (Figure 5). All contents are calculated based on raw material.

For all samples, a decrease in the amount of glucose in the fiber fraction with increasing severity can be observed. Concurrently, the amount of glucose in the extract increases slightly. These effects are less pronounced in MDF sample A and B. Glucose, besides being the main constituent of cellulose, also occurs as a component in hemicelluloses in different amounts, depending on plant species, development stage, and cell wall type [86]. As cellulose is resistant to hydrolysis in hydrothermal treatments at temperatures below 200 °C [82], the liberated glucose likely originates mainly from hemicelluloses. An increase in treatment severity also leads to a reduction in xylose content in the fiber fraction and an increase in the extract fraction for all samples, as the solubilization of the xylose increases. The same can be observed for mannans in poplar and spruce wood. In contrast to that, the mannose content in the fiber and extract fraction of the MDF samples shows almost no changes. This reduced degradation of mannose-containing polysaccharides (glucomannans and galactoglucomannans) might be due to differences in pH and acid content (see Figure 4). This could be an explanation for the differing yield behavior of MDF sample B (Figure 1d), which has comparatively high mannose content, and the reduced removal of glucose from the fiber fraction in MDF samples mentioned before. The amount of glucose, xylose, and mannose found in the extract is less than the corresponding amount of monomers missing in the fiber fraction likely due to degradation. A slight increase in total AIR (sum of extract and fiber fraction AIR) can be observed with increasing severity. The highest total AIR for the poplar, MDF A, and MDF B sample is found at the highest severity of 3.95 with a total AIR of 25.8%, 24.9%, and 29.3%, respectively. For spruce, the highest AIR is found at treatment conditions of 170 °C and 20 min with 29.7%. In comparison to the AIR of the corresponding raw material, this translates to an increase between 2.8% to 5.8%. This increase can be explained with the formation of pseudo-lignin from degradation products of carbohydrates [87,88,89].

Comparing the pH of the extracts, distinct differences between MDF and pure wood samples can be observed. The pH of the poplar extracts decreases from 6.9 to 4.4, while the pH of the spruce extracts stays around 5 in the examined severity range. In general, an acidic milieu is to be expected, as acetic acid is freed from acetyl groups of the wood in hot and humid conditions like they are found in steam treatments [85,90]. In the extracts of waste MDF, on the other hand, the pH rises from 7.6 for MDF A and 7.4 for MDF B at the lowest severity of 2.47 to approximately 8 at higher severities. The high pH of the MDF samples likely originates from ammonium hydroxide, which is converted from ammonia in watery solutions. The ammonia, in turn, is hydrolytically cleaved from the UF-resin polymers [18,19]. For all samples, the amount of formic and acetic acid rises with increasing severity. Despite the lower pH of the wood samples, the amount of formic and acetic acid found is less than those in the MDF extracts. The high acid content in the MDF extracts might be due to reactions of the wood polymers with mineral acids (H_2_SO_4_) released from hardeners ((NH_4_)_2_SO_4_) used in MDF production [20]. Especially in MDF sample A, high amounts of acids can be observed, which could be due to the postulated high amount of hardwood used in the production of MDF sample A and its inherent, in comparison to softwood, high amount of acetyl groups.

Furfural and 5-HMF are compounds resulting from degradation processes of pentoses and hexoses, respectively, under acidic conditions [91,92]. The furfural and 5-HMF content increases with the treatment severity for poplar and spruce wood (Figure 5). By comparison, no degradation products could be found in the MDF samples. Only at the highest severity of 3.95 and only in MDF sample B, a slight rise in amount of degradation products could be observed. Even though the low amount of degradation products might be due to further degradation to formic acid, it is more likely that 5-HMF and furfural are not formed at all during the steam treatment of the MDF samples, as all the aforementioned degradation processes are reported to happen in acidic conditions and are non-existent in alkaline treatments [82].

### 3.5. Observations on Morphological Changes of the Fibrous Material

**Figure 6:**
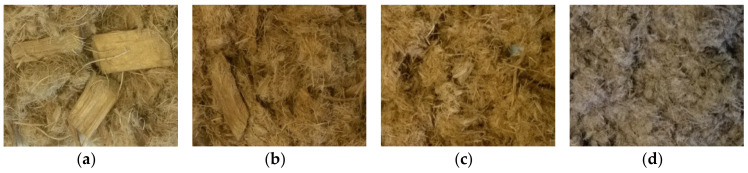
Morphology of poplar wood after steam refining using different treatment conditions [log*R*_0_; °C; min]: (**a**) 2.47; 150; 10, (**b**) 3.06; 160; 20, (**c**) 3.36; 180; 10, (**d**) 3.95; 190; 20.

**Figure 7:**
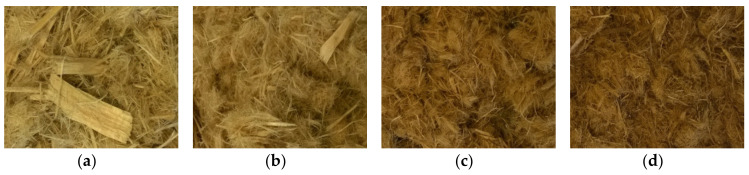
Morphology of spruce wood after steam refining using different treatment conditions [log*R*_0_; °C; min]: (**a**) 2.47; 150; 10, (**b**) 3.06; 160; 20, (**c**) 3.36; 180; 10, (**d**) 3.95; 190; 20.

Because of the intense mechanical forces applied to the MDF and native wood samples in steam refining, changes in the fiber morphology can be expected in addition to the addressed chemical changes. Before the treatment, the sample materials are present as chips, as described in Section 2.1. After a steam-refining treatment at a low severity of 2.47, whole pieces of wood chips can still be found in the fibrous material of the native wood sample (Figure 6a and Figure 7a). At a severity of 3.06, the wood chips are smaller and frayed (Figure 6b and Figure 7b). At higher severities of 3.36 and more, most of the fibrous material is present in small fiber strands and bundles (Figure 6c,d and Figure 7c,d). This is likely due to the lignin softening at the temperature of 165 °C [93], enabling an easier separation of the fibrils at higher severities.

**Figure 8:**
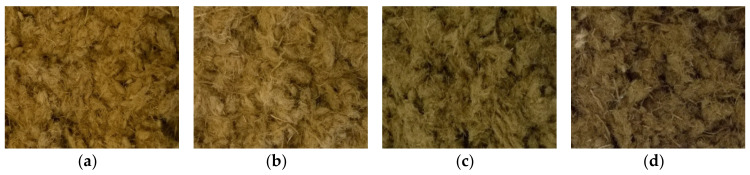
Morphology of MDF sample A after steam refining using different treatment conditions [log*R*_0_; °C; min]: (**a**) 2.47; 150; 10, (**b**) 3.06; 160; 20, (**c**) 3.36; 180; 10, (**d**) 3.95; 190; 20.

The fibers of MDF sample A, on the other hand, are largely separated, independent of treatment severity (Figure 8a–d). MDF sample B (not pictured) shows the same morphology pattern as MDF sample A. Even in steam-refining treatments conducted at the lowest severity grade of 2.47, only small fiber bundles are visible (Figure 8a). This is likely due to fibers already having been separated in the MDF production process and the resin holding the fibers together being dissolved even at the lowest examined severity grade. No distinct changes in the amount or size of the small fiber bundles can be observed at higher severity treatments (Figure 8b–d). Thus, the fibrous fractions remaining in the form of small fiber bundles for all materials are likely due to the fiber bundles being too small for the refining gap in the steam-refining reactor.

An additional mild refiner treatment at low energy input was performed for all samples in order to achieve a complete fiber separation (see Section 2.4). In Table 4, the measured fiber lengths and widths for selected materials are presented and compared to recycled pulp from an industrial corrugated cardboard producer.

By comparing the same raw materials treated at different severities, no clear changes in fiber length and width can be observed. The only exception is the poplar sample treated at 150 °C and 10 min, displaying a distinctively lower average fiber length. This might be due to an increase in number of small fibers, as the fiber sample included almost intact wood chips after steam refining. The insufficient softening of the structure might lead to increased rupture of the fibers in the refiner and a higher amount of small fibers in comparison to the poplar samples treated at higher severities.

However, distinct differences between the different samples dependent on the raw material can be observed. As stated in the literature, with a fiber length of 1.7 to 4.6 mm, untreated spruce fibers (*Picea abies*) are longer than untreated poplar fibers (*Populus spp*.) with fiber lengths from 0.6 to 1.6 mm [81]. After the steam and refining treatment, the spruce fibers with average lengths of 0.89 to 0.97 mm are still longer than the poplar fibers with average fiber lengths of 0.68 to 0.87 mm. However, as the size reduction is much greater for the spruce fibers, the average fiber lengths converge. In MDF sample A, with measured average fiber lengths of 0.79 to 0.86 mm, the fibers are shorter than those in MDF sample B, with average fiber lengths ranging from 0.95 to 1.02 mm. In addition, the fibers from MDF A show smaller average widths of roughly 25 mm in comparison to the fibers from MDF sample B with average widths of 29.4 to 30.3 mm. Thus, the widths of MDF sample A are closer to the widths of the poplar samples, while the widths of MDF sample B are close to the widths of the spruce samples. These results support the assumption of a higher softwood content in MDF sample B, as fibers from softwoods usually have larger dimensions than fibers from hardwoods. Surprisingly, the spruce fibers are shorter than the fibers of MDF sample B. This could be due to the lignocellulosic material used in the production of MDF sample B, which might have included softwood with even longer fibers. Another possible explanation is, as the fibers have been separated before in the MDF production process, the MDF fibers experience less mechanical forces in the refining, leading to longer fibers. As the fibers from MDF B are close to the fiber length of the recycled pulp for packaging applications, they might be acceptable as an added reinforcement fiber in such applications.

## 4. Conclusions

With steam refining, it is possible to gain an extract containing up to 30% of carbohydrates while still retaining a high yield fiber fraction for further conversion from post-consumer MDF. As the fibers are brownish in color, they might be useable in packaging paper or for re-use in MDF production. The extract is a mixture of different hemicelluloses and lignin residues. Such crude mixtures show promising stabilization properties as oil-in-water emulsifiers [44] and might be useable in such applications.

The MDF samples show a distinctively different behavior in steam treatments to conventional spruce and poplar wood. Roughly 80% of the nitrogen compounds is removed from the fibers and can be found either in the extract or in the gaseous phase, depending on the treatment severity. The hydrolysis and subsequent solubilization of the resins lead to an increase in acid content and pH of the condensed steam (i.e., extract). The changed milieu seems to hamper the hydrolysis of the lignocellulosic polymers. At the same time, for steam treatment characteristics, the degradation products from monomers such as furfural and 5-HMF could not be detected. In both MDF samples, no significant differences between experiments performed at the same severity resulting from different treatment parameter combinations could be observed. Thus, the severity factor used for steam treatment of wood seems to be suitable for the evaluation of steam treatments of waste MDF. This also means that, depending on local economic factors such as unit labor cost and energy prices, a tradeoff between reaction time and temperature can be made. Future investigations may focus on the potential of the fibers for packaging applications and the utilization of hemicelluloses from the extract fraction.

## Figures and Tables

**Table 1:** Experimental plan with reaction conditions.

Experimental Run	Temperature	Duration	Severity
#	°C	min	log*R*_0_
1	150	10	2.47
2	150	20	2.77
3	160	10	2.77
4	160	20	3.07
5	170	10	3.06
6	170	20	3.36
7	180	10	3.36
8	180	20	3.66
9	190	10	3.65
10	190	20	3.95

**Table 2:** Extract, carbohydrate, lignin, nitrogen, and ash contents of poplar, spruce, and medium-density fiberboard (MDF) sample A and B in [%] based on raw material.

Raw Material		Poplar spp.	Spruce spp.	MDF A	MDF B
Extracts	Petroleum-ether	0.2	0.3	0.7	0.9
	Acetone/H_2_O (9:1)	1.6	0.9	4.8	4.4
	H_2_O	1.1	0.6	7.0	10.5
	∑	3.0	1.9	12.5	15.8
Carbohydrates	Glucose	48.3	48.3	38.3	37.6
	Xylose	14.0	5.6	12.4	5.7
	Mannose	2.7	12.7	4.0	7.2
	Galactose	0.5	1.8	0.7	1.3
	Arabinose	0.3	1.0	0.4	0.5
	Rhamnose	0.3	0.1	0.2	0.1
	∑	66.0	69.5	56.0	52.3
Residue	Acid soluble	2.5	1.2	2.3	0.9
	Acid insoluble	20.0	25.7	22.1	24.6
	∑	22.5	26.9	24.4	26.6
Nitrogen content	Before ASE	0.3	0.3	4.2	4.4
	After ASE	0.2	0.2	1.4	0.9
Ash		0.9	0.3	0.6	0.5

**Table 3:** Carbohydrates and lignin content in the fiber and extract fraction (in % based on raw material).

			Fiber Fraction					Extract Fraction				
	Run	Severity	Glc	Xyl	Man	OC *	AIR	Glc	Xyl	Man	OC *	AIR
	#	log*R*_0_	[%]	[%]	[%]	[%]	[%]	[%]	[%]	[%]	[%]	[%]
Poplar	1	2.47	48.2	14.0	2.3	1.0	23.8	0.1	0.1	0.2	0.2	0.6
	2	2.77	47.4	13.3	2.2	0.8	23.5	0.1	0.1	0.2	0.2	0.8
	3	2.77	47.1	13.4	2.3	0.9	23.3	0.1	0.1	0.2	0.3	0.7
	4	3.07	47.0	13.2	2.2	0.8	22.8	0.1	0.4	0.3	0.4	0.6
	5	3.06	48.0	13.0	2.1	0.8	23.6	0.1	0.8	0.4	0.5	0.8
	6	3.36	46.7	9.1	2.0	0.4	23.7	0.3	4.5	0.5	0.7	1.1
	7	3.36	49.1	9.4	1.8	0.5	23.1	0.3	3.4	0.5	0.7	1.0
	8	3.66	47.1	7.9	1.8	0.3	24.2	0.3	6.2	0.7	0.8	1.0
	9	3.65	46.4	6.3	1.6	0.3	24.1	0.3	7.3	1.0	0.8	0.9
	10	3.95	46.5	4.1	1.3	0.1	24.5	0.3	7.6	1.0	0.8	1.3
Spruce	1	2.47	44.2	4.8	11.7	2.3	29.1	0.2	0.0	0.3	0.4	0.3
	2	2.77	43.9	4.7	11.2	1.9	27.9	0.2	0.1	0.6	0.7	0.5
	3	2.77	43.8	4.6	10.9	1.8	27.7	0.2	0.1	0.8	0.7	0.4
	4	3.07	45.0	4.7	10.7	1.7	28.3	0.3	0.3	1.5	1.1	0.3
	5	3.06	41.1	4.2	9.5	1.4	28.6	0.4	0.5	2.0	1.1	0.3
	6	3.36	42.7	4.0	7.4	1.1	29.3	0.6	1.0	3.5	1.4	0.4
	7	3.36	42.9	3.7	7.0	0.8	27.6	0.7	1.3	4.6	1.5	0.1
	8	3.66	40.0	3.0	4.8	0.5	28.4	1.1	1.9	6.5	1.8	0.4
	9	3.65	40.2	2.6	4.3	0.4	27.5	0.9	2.0	6.8	1.7	1.0
	10	3.95	41.6	2.3	2.9	0.2	26.5	1.0	2.1	8.2	1.7	0.9
MDF A	1	2.47	36.1	11.9	3.7	1.1	23.2	0.2	0.4	0.3	0.4	0.7
	2	2.77	36.1	11.8	3.6	1.0	22.4	0.2	0.5	0.3	0.4	0.8
	3	2.77	35.7	11.5	3.6	0.9	21.0	0.2	0.7	0.3	0.4	1.7
	4	3.07	35.6	10.9	3.7	0.9	22.0	0.3	1.0	0.3	0.5	1.1
	5	3.06	35.4	10.7	3.9	0.8	22.5	0.2	1.0	0.2	0.4	1.6
	6	3.36	34.2	9.8	3.3	0.6	21.0	0.3	2.6	0.2	0.5	2.6
	7	3.36	33.4	9.6	3.5	0.7	22.8	0.3	2.2	0.2	0.5	1.0
	8	3.66	34.1	8.0	3.5	0.6	22.4	0.3	3.8	0.1	0.5	2.1
	9	3.65	35.5	7.5	3.4	0.5	21.7	0.3	4.3	0.1	0.5	2.2
	10	3.95	35.2	5.8	3.4	0.4	24.0	0.3	4.7	0.2	0.5	0.9
MDF B	1	2.47	38.3	5.7	7.0	1.8	25.9	0.6	0.5	1.1	1.0	2.1
	2	2.77	38.4	5.6	6.9	1.7	26.4	0.6	0.6	1.2	0.9	1.8
	3	2.77	38.0	5.4	6.8	1.8	26.2	0.6	0.7	1.2	0.9	2.0
	4	3.07	37.8	5.3	6.8	1.7	26.9	0.6	0.9	1.1	0.9	2.0
	5	3.06	37.9	5.2	6.7	1.5	26.6	0.6	0.9	1.2	0.9	2.0
	6	3.36	38.6	4.9	6.9	1.7	25.9	0.6	1.3	1.0	0.9	2.1
	7	3.36	39.3	4.8	6.9	1.6	26.3	0.6	1.4	1.1	0.9	2.1
	8	3.66	39.3	4.3	6.9	1.6	26.2	0.6	1.9	1.1	0.9	2.3
	9	3.65	37.9	4.0	6.5	1.5	27.0	0.6	2.0	1.1	0.9	2.1
	10	3.95	37.7	3.3	6.2	1.3	26.9	0.6	2.3	1.2	0.9	2.4

* Other carbohydrates (arabinose, rhamnose, galactose).

**Table 4:** Fiber length and diameter of steam-treated poplar, spruce, MDF A, MDF B, and recycled pulp.

Sample	Severity	Temperature	Duration	Fiber Length	Diameter
	[log*R*_0_]	[°C]	[min]	[mm]	[µm]
Poplar	2.47	150	10	0.68	25.6
Poplar	3.36	170	20	0.87	24.4
Poplar	3.95	190	20	0.80	22.7
Spruce	2.47	150	10	0.90	28.3
Spruce	3.36	170	20	0.97	28.6
Spruce	3.95	190	20	0.89	28.5
MDF A	2.47	150	10	0.82	25.0
MDF A	3.36	170	20	0.86	25.5
MDF A	3.95	190	20	0.79	24.3
MDF B	2.47	150	10	1.02	30.3
MDF B	3.36	170	20	0.95	29.9
MDF B	3.95	190	20	0.95	29.4
Recycled pulp	-	-	-	1.09	22.1

## Appendix A

**Table A1:** Fiber and extract yield of poplar, spruce, and MDF sample A and B at treatment severities from 2.47 to 3.95.

	Run	Severity	Temperature	Duration	Fiber Yield	Extract Yield
	#	[log*R*_0_]	[°C]	[min]	[%]	[%]
Poplar	1	2.47	150	10	96.1	3.3
	2	2.77	150	20	93.7	3.2
	3	2.77	160	10	94.5	3.6
	4	3.07	160	20	92.3	4.2
	5	3.06	170	10	93.4	5.3
	6	3.36	170	20	84.7	11.5
	7	3.36	180	10	87.4	10.5
	8	3.66	180	20	83.9	14.3
	9	3.65	190	10	80.4	16.5
	10	3.95	190	20	77.4	18.1
Spruce	1	2.47	150	10	95.9	1.9
	2	2.77	150	20	93.7	3.1
	3	2.77	160	10	92.6	3.4
	4	3.07	160	20	92.0	6.0
	5	3.06	170	10	89.2	6.2
	6	3.36	170	20	88.0	10.4
	7	3.36	180	10	83.9	12.1
	8	3.66	180	20	79.9	16.5
	9	3.65	190	10	77.4	17.5
	10	3.95	190	20	72.6	19.6
MDF A	1	2.47	150	10	85.3	12.1
	2	2.77	150	20	84.6	12.8
	3	2.77	160	10	82.4	13.7
	4	3.07	160	20	82.9	14.8
	5	3.06	170	10	81.3	13.3
	6	3.36	170	20	77.3	17.4
	7	3.36	180	10	78.6	16.2
	8	3.66	180	20	74.9	16.7
	9	3.65	190	10	73.3	19.5
	10	3.95	190	20	72.9	18.5
MDF B	1	2.47	150	10	82.0	18.4
	2	2.77	150	20	82.1	18.1
	3	2.77	160	10	81.5	18.6
	4	3.07	160	20	81.2	17.6
	5	3.06	170	10	81.1	17.5
	6	3.36	170	20	79.7	17.0
	7	3.36	180	10	79.8	17.4
	8	3.66	180	20	77.9	17.9
	9	3.65	190	10	77.8	17.7
	10	3.95	190	20	76.1	18.2
